# Tatton-Brown-Rahman syndrome with a novel DNMT3A mutation presented severe intellectual disability and autism spectrum disorder

**DOI:** 10.1038/s41439-020-0102-6

**Published:** 2020-05-18

**Authors:** Takayuki Yokoi, Yumi Enomoto, Takuya Naruto, Kenji Kurosawa, Norimichi Higurashi

**Affiliations:** 1 Department of Pediatrics, The Jikei University School of Medicine, Tokyo, Japan; 2 Division of Medical Genetics, Kanagawa Children’s Medical Center, Yokohama, Japan; 3 Clinical Research Institute, Kanagawa Children’s Medical Center, Yokohama, Japan; 4 Pediatrics and Developmental Biology, Tokyo Medical and Dental University Graduate School, Tokyo, Japan

**Keywords:** Paediatric neurological disorders, Development

## Abstract

Tatton-Brown-Rahman syndrome is a congenital anomaly syndrome that manifests with overgrowth, macrocephaly, and characteristic facial features. This autosomal dominant disease is caused by a germline mutation in *DNMT3A*. Some patients with this syndrome develop mild to severe intellectual disability, which is sometimes accompanied by autism spectrum disorder or other developmental disorders. We report a Japanese patient with severe intellectual disability and autism spectrum disorder with a de novo mutation in the active domain of *DNMT3A*.

There are several congenital anomaly syndromes that exhibit overgrowth. Tatton-Brown-Rahman syndrome (TBRS) was clinically reported together with the responsible gene *DNMT3A* in 2014 and is a relatively new overgrowth congenital anomaly syndrome^1^. *DNMT3A*: DNA methyltransferase 3 alpha is involved in DNA de novo methylation essential for genome regulation and development. In addition to overgrowth, patients with TBRS also have macrocephaly, dysmorphic facial features and intellectual disability (ID) with or without autism spectrum disorder (ASD). TBRS is inherited in an autosomal dominant manner; most of the mutations are de novo mutations^1–6^. To date, there are approximately 80 reported cases^1–8^. Various mutations have been found at various sites in previous reports of TBRS. Originally, somatic mutations of *DNMT3A* were identified in acute myeloid leukemia. There have been several reports of TBRS with the same mutation as acute myeloid leukemia^3–6^. ID varies from mild to severe, and it can be combined with developmental disorders. Here, we report a case of TBRS with a novel mutation and severe ID and ASD.

The proband was a 3-year-old Japanese boy born to nonconsanguineous healthy parents. He was born at 37 weeks of gestation with a birth weight of 3630 g (+3.2 S.D.), a length of 98.1 cm (+1.8 S.D.), and head circumference of 35.5 cm (+2.1 S.D.). His weight was 23.5 kg (+2.3 S.D.), his height was 117 cm (+2.4 S.D.), and his head circumference was 54.5 cm (+4.2 S.D.) at 5 years of age. Although his motor developmental milestone was within normal limits, his intellectual developmental milestone was delayed. He began to speak a single word at 2 years and 8 months of age and could speak several words at 3 years. He could follow only very easy and simple instructions. He developed complex febrile seizures at the age of 2 years. Physical examination revealed dysmorphic features, including narrow forehead, synophrys, hypertelorism, depressed nasal bridge, and an upturned nose (Fig. 1a). His brain MRI showed no abnormal findings. Because hyperactivity and autistic behavior were remarkable, it was difficult to carry out any developmental testing. His karyotype analysis was 46,XY, and deletion of 5q35 was not detected by fluorescence in situ hybridization (FISH) analysis.

**Fig. 1:**
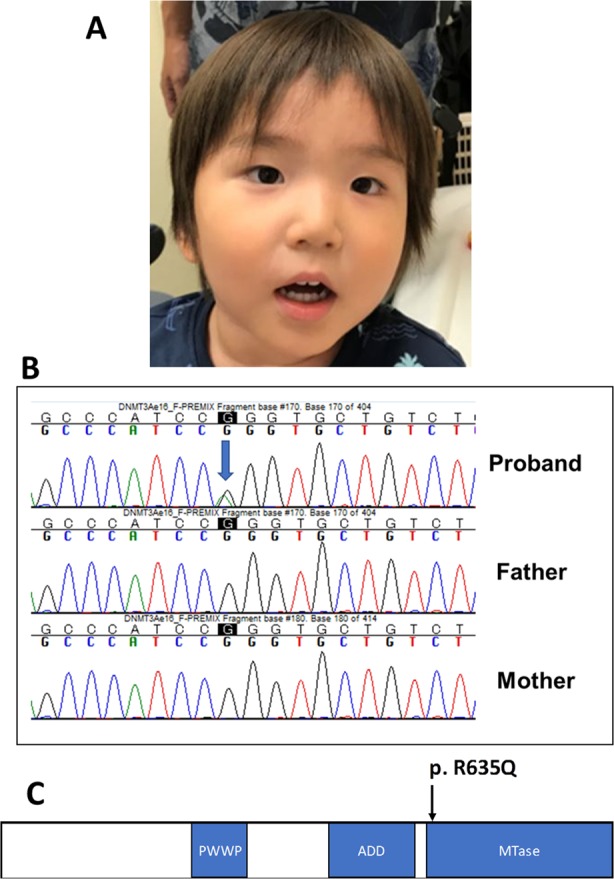
The clinical features and the pathogenic variant in *DNMT3A* of this patient. **a** Photograph of the patient at 3 years of age, which was permitted to be published in any journal by his parents. The patient had a rounded face, narrow forehead, synophrys, hypertelorism, depressed nasal bridge, and an upturned nose. **b** The mutation in *DNMT3A* was identified in the patient by targeted sequencing. Sanger sequencing demonstrated that the mutation occurred de novo. **c** The *DNMT3A* structure and mutation of our patient. PWWP proline-tryptophan-tryptophan-proline, ADD ATRX-Dnmt3-Dnmt3L; Mtase methyltransferase.

Written informed consent was obtained from the parents of the patients in accordance with the Kanagawa Children’s Medical Center Review Board and Ethics Committee.

Total genomic DNA was obtained from lymphocytes using the QIAamp DNA Blood Mini Kit (Qiagen, Valencia, CA, USA) following the manufacturer’s instructions.

DNA libraries were enriched for sequences using TruSight One (Illumina Inc., San Diego, CA, USA), which enables enrichment and final analysis of a panel of 4,813 genes. Patient samples were sequenced by MiSeq (Illumina Inc.) with 150-bp paired-end reads. Data were analyzed using the Burrows–Wheeler alignment tool and the Genome Analysis Toolkit pipeline (Broad Institute, Cambridge, MA, USA) and visualized in the Integrative Genomics Viewer (IGV). Calling copy-number variation (CNV) was based on log–ratio analysis and read depth *z*-score of each exon. Mutations identified by targeted sequencing were confirmed by Sanger sequencing, and appropriate segregation was demonstrated by phenotype in the unaffected parents.

Targeted sequencing identified a novel mutation, c.1904G>A: p.R635Q (NM_175629), in *DNMT3A* (Fig. 1b). The mutation was absent in a human genetic variation database (the Japanese genetic variation consortium: a reference database of genetic variations in the Japanese population comprising 1208 individuals [http://www.genome.med.kyoto-u.ac.jp/SnpDB]), the 1000 Genomes project, the National Heart, Lung, and Blood Institute (NHLBI) grant opportunity exome sequencing project (ESP), the Exome Aggregation Consortium (ExAC), and our 600 in-house control Japanese genomic samples. In silico analysis according to ANNOVAR, with predictions for c.1904G>A (p.R635Q), indicated a deleterious effect by SIFT (http://sift.jcvi.org/), Polyphen-2 (http://genetics.bwh.harvard.edu/pph2/), and MutationTaster (http://neurocore.charite.de/MutationTaster/). Sanger sequencing demonstrated that the mutation was de novo.

Although it was a missense mutation, the phenotype could be more distinctive and severe due to the mutation of the active domain. In this case, the ID was severe. We could not derive the relationship between the severity of mental retardation and the position/type of mutation either from this study or in comparison to previous reports (Fig. 2a, b)^1–6^. Our patient also displayed autistic behavior. Similarly, the relationship between the presence or absence of autism and the position and type of mutation is unknown. Additionally, the relationship between ID and ASD is unknown (data not shown).

**Fig. 2:**
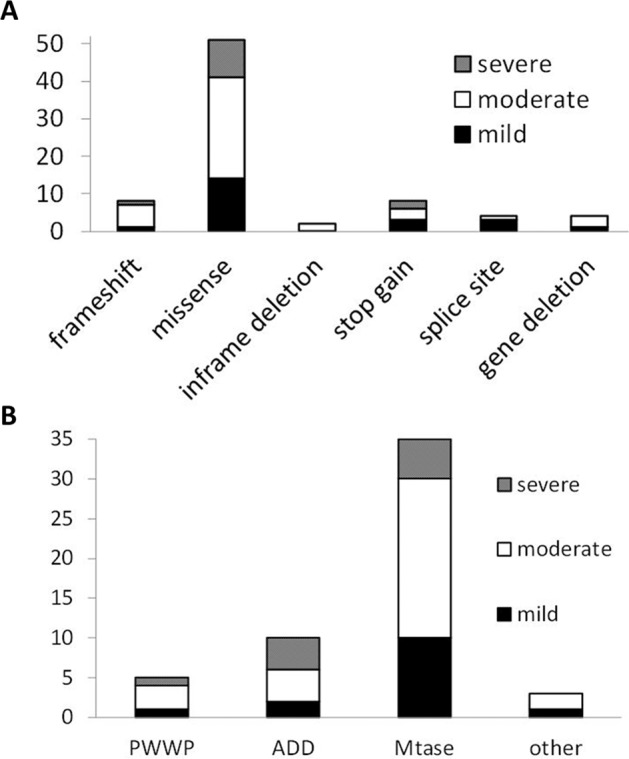
Correlation between genotypes of *DNMT3A* and phenotypes. **a** Correlation between variant types of *DNMT3A* and phenotypes. **b** Correlation between locus of mutations in *DNMT3A* and severity of intellectual disability. The vertical axis indicates the number of patients. PWWP proline-tryptophan-ryptophan-proline, ADD ATRX-Dnmt3-Dnmt3L, Mtase methyltransferase.

In some congenital anomalies that exhibit overgrowth, such as TBRS, tumorigenesis may be seen. Another mutation in *DNMT3A*, p.R882H, in its active domain, has been identified as somatic mutations in leukemic cells. One patient with this mutation developed acute myeloid leukemia^4^. In our patient, the mutation was in the active domain. However, no onset of leukemia or other blood abnormalities was observed in our patient. Although it is unknown whether this mutation leads to leukemia, it is important to screen for the onset of leukemia in the future.

In conclusion, we presented a patient with TBRS with a novel mutation. He had severe ID and ASD. Since the severity of ID, ASD, and leukemia is significantly related to the prognosis and quality of life, if the genotype-phenotype correlation is known, it is very useful for patients and their families. It is necessary to accumulate future cases.

## Data Availability

The relevant data from this Data Report are hosted at the Human Genome Variation Database at 10.6084/m9.figshare.hgv.2844.
